# Practices Supporting Community Recovery and Healing from Climate-Related Disasters: A Systematic Review

**DOI:** 10.3390/ijerph21060795

**Published:** 2024-06-18

**Authors:** Lynne Keevers, Deborah Gough, Jacqui Cameron, Anthony McKnight, Saskia Ebejer, Susan Duchesne, Adam Gowen, Karen Fildes, Maria Mackay

**Affiliations:** 1 School of Health Sciences, Faculty of Health, University of Canberra, Canberra, ACT 2617, Australia; lynne.keevers@canberra.edu.au; 2 School of Health and Society, Faculty of the Arts, Social Sciences and Humanities, University of Wollongong, Wollongong, NSW 2522, Australia; 3 Deputy Vice-Chancellor, Academic & Student Life, Bega Valley Campus, University of Wollongong, Bega, NSW 2550, Australia; gough@uow.edu.au; 4 Department of Social Work, The University of Melbourne, Carlton, VIC 3010, Australia; 5 UOW Indigenous Strategy Unit, University of Wollongong, Wollongong, NSW 2522, Australia; anthonym@uow.edu.au; 6 Faculty of the Arts, Social Sciences and Humanities, Eurobodalla Campus, University of Wollongong, Batemans Bay, NSW 2536, Australia; sebejer@uow.edu.au; 7 School of Education, Faculty of Arts, Social Sciences and Humanities, Bega Valley Campus, University of Wollongong, Bega, NSW 2550, Australia; sued@uow.edu.au; 8 Country Universities Centre Southern Shoalhaven, Ulladulla, NSW 2539, Australia; adam.gowen@cucsouthernshoalhaven.edu.au; 9 School of Medical, Indigenous and Health Science, Faculty of Science, Medicine and Health, University of Wollongong, Wollongong, NSW 2522, Australia; kfildes@uow.edu.au; 10 Ngarruwan Ngadju First Peoples Health and Wellbeing Research Centre, University of Wollongong, Wollongong, NSW 2522, Australia; mmackay@uow.edu.au

**Keywords:** disasters, healing, practices, recovery, community, systematic review

## Abstract

This systematic review investigated the practices that support and hinder the recovery and healing of communities and the environments within which they live, following climate-related environmental disasters. Although the literature focused on recovery is dominated by interventions aimed at the individual and their mental health, a thematic analysis of thirty-six studies established a range of practices that enhance collective recovery and healing. Four narratives were identified from the findings highlighting key practices: (1) collective and community-led recovery; (2) recognising the criticality of context, place, and identity; (3) adopting a holistic conception of well-being and Country-centred practices; and (4) decolonising and Indigenising the literature. This study details recommendations for research and practice. First Nations’ knowledges and healing practices need to be recognised and harnessed in climate-related environmental disaster recovery. Community-led interventions harness local knowledge, networks, and expertise, which improves the dissemination of resources and enables recovery efforts to be tailored to the specific needs of communities.

## 1. Introduction

Climate-related disasters such as droughts, bushfires, cyclones, and floods are increasing in prevalence and severity around the globe [1,2]. Disasters generate devastating impacts on the physical, social, and emotional well-being of individuals, communities, and the environments in which they live [3]. The 2019/2020 Black Summer bushfires followed by floods on the Southeast Coast of Australia were without parallel in their ferocity and spread. The Southeast Coast, already struggling with extreme dryness, experienced unrelenting wildfires in which eighty percent of the region was burnt, and more than 800 million animals died [4,5]. People were also killed, and habitats, homes, and other structures were destroyed in these rural and regional communities [6]. The wildfires were finally extinguished by heavy rains culminating in floods, landslides, and landslips. Conditions of consecutive disasters have been coined permacrisis, with some scholars arguing that we are living in a volatile ‘Anthropocene’ marked by human-caused [7] burdens that produce permanent ruptures to the Earth’s climate, environment, and ecosystems [8]. In such times of permacrisis, there is an urgent need to be well prepared to assist community recovery and healing post-disaster. Accordingly, as part of a broader research project, an interdisciplinary team of Aboriginal and non-Aboriginal researchers reviewed the international literature to identify the practices and interventions that support and/or hinder healing and recovery for communities impacted by climate-related, environmental disasters. The broader project uses a Country-led and practice-based theoretical framework to co-design methodology and data collection methods that are respectful, contribute to healing and recovery for research participants with the lived experience of the South Coast bushfires and foreground the Aboriginal concept of Country. Accordingly in this review, Country is capitalised as it is not only a common noun but also a proper noun. As discussed by Rose [9] (p. 7), ‘people talk about Country in the same way that they would talk about a person: they speak to Country, sing to Country, visit Country, worry about Country, feel sorry for Country, and long for Country. People say that Country knows, hears, smells, takes notice, cares, and is sorry or happy. Country is a living entity with a yesterday, today, and tomorrow, with a consciousness and a will toward life’. Specifically, this systematic review addresses the following question: What is the evidence about practices and interventions that support or hinder healing post-climate-related disasters for communities and the environments within which they live?

To answer this question, we included studies that discuss practices and/or interventions focusing on the ‘doings, sayings, relatings’ and the spiritual and material arrangements [10] evident in the literature that contribute to and/or restrain healing and recovery post-disasters.

This review adopts an Aboriginal-informed Country-centred notion of community that is inclusive of all entities: water, air, land, flora, fauna, insects, humans, and the entangled relations amongst them. Additionally, the research team includes both insider and outsider researchers [11], as many of us were motivated to undertake this research following our own experiences of the South Coast bushfires.

This review acknowledges that defining disasters is complex and that no unified definition of disaster exists [12]. Quarantelli’s ([13] (p. 682) seminal definition characterises disasters as having a number of defining features including that they are sudden-onset occasions that seriously disrupt the routines of collective units; cause the adoption of unplanned courses of action to adjust to the disruption; have unexpected life histories designated in social space and time; and pose danger to valued social objects and represent a vulnerability that reflects weaknesses in social systems ([14] (p. 345). This theoretical approach, which foregrounds a social conception of disasters, offers a good fit for the broader project, which is underpinned by social practice theory. The broader project also guided us to restrict our search to climate-related environmental disasters, thereby excluding articles examining humanitarian crises, war, conflicts, infectious diseases/pandemics, or industrial or nuclear accidents, the latter of which is included in some global definitions of environmental hazards, for example, the Sendai Framework for Disaster Risk Reduction 2015–2030 [15]. Whilst we acknowledge a loose association with our work in the Sendai Frameworks’ Action Priority 4, which refers to enhancing preparedness as pivotal to recovery, the alignment is not such to warrant including this extended definition in our paper, bounded as it is by a broader project with a strict focus on climate-related environmental disasters. Hollis ([8] (p. 302) maintains that within disaster studies, ‘a central idea manifest in most definitions is one of disruption and a return to a state of normalcy’. However, as Hollis points out, conceptualising disaster in the Anthropocene disrupts these notions of a return to normalcy or stability. Further, it fundamentally questions assumptions of human exceptionalism which position the human species as apart from our responsibilities to the other lives that constitute our local and global worlds [16]. This conception that does not view ‘human nature as an interspecies relationship’ ([17] (p. 141), but instead separates humans from nature, is evident in Quarantelli’s [2000] definition above, the Sendai Framework and is common in the disaster literature.

## 2. Materials and Methods

### 2.1. Search Strategy

The protocol for this review was registered with PROSPERO (CRD42022299621). Five databases were searched including Scopus (377), Medline (340), PsycINFO (8980, CINAHL (1093), and Social Science Database (840). These searches were supported by reference checking of included studies, forward citations, and consultation with field experts. The search used subject headings, text words, and keywords for practice/intervention/healing; natural disaster; and community/environment. An example of a search string is provided in Table 1.

### 2.2. Inclusion and Exclusion Criteria

We included studies published between 2002 and 2022. All study designs were included: quantitative (*n* = 3), qualitative (*n* = 26), and mixed-method (*n* = 7). The heterogeneity of the included studies meant we could not conduct any metanalysis. A detailed table (see Table 2) presents the structured inclusion/exclusion criteria that were imported to Covidence for screening [18]. Studies included focused on community and reported on practices that supported healing post-disasters triggered by natural hazards.

### 2.3. Selection of Studies

The web-based tool Covidence [18] was used to manage citations during the review process. All titles and abstracts were independently screened initially by two team members. Any duplicates were then removed. Studies determined to be possibly relevant or whose eligibility was not clear were retrieved for full-text review. Three team members then independently assessed all full-text articles. Any conflicts were discussed by the three team members.

### 2.4. Data Extraction and Analysis

We modified the Covidence template for data extraction [18]. The data for each study included year, title, authors, abstract, target population, country, study characteristics, study design, participants, intervention, outcomes, and preliminary coding. Data were extracted by all members of the team. A coding tree was developed by all team members, after reading a selection of three papers and agreeing on initial codes, these codes were adapted and added to Covidence for further development during data extraction and later the analysis. After data extraction, the data were then exported to Excel for further analysis. All members of the review team were involved in coding and developing the themes using a thematic approach as described by Braun & Clarke [19]. The final codes and themes were checked with all team members to ensure they were an accurate depiction of the data extracted.

### 2.5. Quality Appraisal

The quality appraisal was undertaken using the Mixed-methods Appraisal Tool (MMAT) [20], which provides support for appraising studies that include a range of study methods and designs.

## 3. Results

We identified 36 studies published between 2002 and 2022 that met the inclusion criteria. The included papers (*n* = 36) represented a range of countries including Australia [21,22,23,24,25,26], Canada [27,28,29,30], Chile [31], China [32,33], India [34], Japan [35,36], New Zealand [37,38,39,40,41], Nepal, Pakistan [42], Samoa [43], United Kingdom, United States [44,45,46,47,48,49,50] and Vanuatu [51].

A summary of the characteristics of the included studies is provided in Table 3.

**Figure 1:**
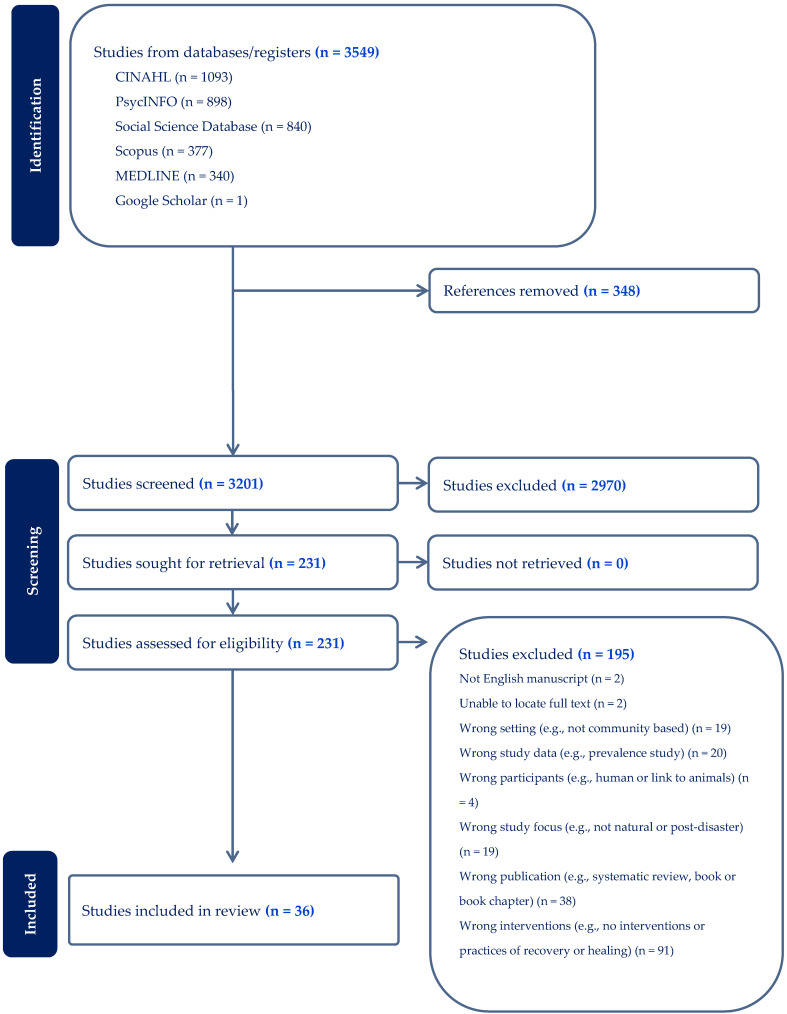
PRISMA chart.

Figure 1 depicts the flow of studies presented following the PRISMA [52] (Preferred Reporting Items for Systematic Reviews and Meta-Analyses) guidelines.

A summary of the quality appraisal undertaken using the MMAT is provided in Table 4.

The findings were divided into six themes. These included (1) practices of belonging and community connectedness, (2) community-led recovery, (3) leading and leadership, (4) restraints to healing and recovery, (5) strength-based approaches, and (6) holistic conception of well-being.

### 3.1. Practices of Belonging and Community Connectedness

Practices of belonging and community connectedness were the most prominent themes, evident in 30 of the 36 studies reviewed. This theme is explored in four sub-themes: a sense of place and belonging interlinked with personal identity; the importance of family and friends’ support for healing and recovery; the role of social capital in disaster recovery; and community formation post-disaster. These practices work together to create a sense of belonging, a sense of place, a sense of safety, and a sense of hope for the future. 

#### 3.1.1. Sense of Place and Belonging Interlinked with Personal Identity

Eleven studies identify a sense of place and belonging interlinked with personal identity as a critical resource in recovery from disasters [22,23,27,29,30,32,40,43,48,51,53]. Tudor and colleagues [40] articulate place as a ‘grounding’ tool, important for re-establishing a safe place of connection. People who have experienced disaster often interlink their recovery identity with a sense of belonging to a place [29]. The importance of returning to everyday pre-disaster practices, customs, and traditions to build belonging was described in two studies [32,51], wherein the importance of designing interventions embedded in cultural practices and community to support healing and recovery is exemplified [43].

A sense of identity is not always connected to a physical place or the built environment. In Stofferahn [48], we learn that it was cultural capital, in the form of the Norwegians’ identity, interlinked with ancestry and anchored in a strong work ethic, that enabled recovery. Lalani and colleagues [27] argue the essentiality of spiritual well-being if people are to heal. In Block and colleagues [23], we observe connectivity, not to physical structures, but to bushland, where the regeneration of the bush reconnected people to place. Ali and colleagues [22] extend this connection to ‘bush’, speaking of the fundamental links that First Nations people have to Country.

#### 3.1.2. Importance of Family and Friends Support for Healing and Recovery

Four studies identify the importance of family and friends’ support as a critical resource in recovery from disasters [21,22,29,43]. In Mohr’s [29] study, a theme of deepened post-disaster interpersonal ties emerges [29]. The significance of family ties is also a consistent refrain, as is a heightened sense of belonging to the larger community. Harms and colleagues [21] acknowledge the importance of formal support structures but, like Tamasese and colleagues [43], argue that the role of family, friends, and community is of utmost importance. Ali and colleagues [22] bring to this discussion an Australian First Nations people’s perspective, arguing that it is kinship and accompanying relationships that support people in their everyday lives and in emergencies.

#### 3.1.3. The Role of Social Capital

Thirteen studies identify social capital as a critical resource in recovery from disasters [21,22,30,33,39,41,42,43,45,47,48,49,53]. Three types of social capital—bonding, bridging, and linking—are identified in the papers. Storr and colleagues [49] describe bonding social capital as the robust connections that exist between people in homogenous groups and bridging social capital as the ‘weak ties that exist between the members of heterogeneous groups’. Further, ‘Linking social capital refers to the connections that exist between individuals from completely different social settings or communities’ [49].

The literature on post-disaster recovery suggests that close, homogenous communities are better positioned than communities marked by diversity and loose connections in relation to recovery, as they have higher levels of bridging and bonding social capital [42,48,49]. Onstad and colleagues [47] argue that building social capital, before and after a disaster, is about local people having a sense of community, belonging, and trust and establishing networks for disseminating information and providing assistance. Recovery activities that support social interaction and connectedness, built on local practices and values, have a significant role in disaster response programs [33,36,45]. Dionisio and Pawson [39] suggest that ‘new communities of action emerge in the wake of disaster, and new bonds of social capital are created as people self-organise to meet immediate needs’. 

Despite the dominant view evident in the literature that social capital is critical to disaster recovery, the practices and interventions that generate social capital before or after disasters are not thoroughly investigated or articulated in the included studies. One paper only, authored by Onstad and colleagues [47], offers some suggestions arguing that ensuring local people are centrally involved in the recovery process and providing them with the resources they need to take ownership of the recovery process promoted social capital following a flood disaster.

#### 3.1.4. Community Formation Post-Disaster

Four studies identify community formation post-disaster as a critical resource in recovery from disasters [21,24,28,51]. Complementing the studies that demonstrate that high levels of pre-disaster social capital are predictive of faster community recovery and rebound, several studies discuss how the shared experience of disaster may generate a greater sense of community and belonging, which was not necessarily evident or a central feature of their lives before the disaster [24,28,51]. Silver and colleagues [28] highlight how feelings of uncertainty, guilt, grief, and loss were co-existent with shared strong feelings of belonging.

Clissold and colleagues [51] note the key role of joining together as a community to bring back cultural and everyday, pre-disaster practices. Scott and colleagues [24] stress the value of putting recovery in the hands of community-directed initiatives that build on community assets and social connections.

### 3.2. Community-Led Recovery

Community-led interventions ranked high in the literature, with 28 articles referring to this aspect of post-disaster responses [22,24,26,27,29,30,31,32,33,35,36,38,39,40,41,43,44,45,46,47,48,49,50,51,53,54,55]. This theme is discussed in three subthemes: the importance of local knowledge, community-based creative practices, and community-led practices. These community-led recovery interventions tended to focus on locally situated, longer-term strategies with an emphasis on healing by rebuilding the community, and thereby creating a sense of belonging and hope.

Rather than focusing only on immediate material needs, community-led interventions often address emotional distress [29,36,46,51], with attention towards longer-term whole-of-community recovery [26,36,47].

#### 3.2.1. Local Knowledge

The usefulness of local knowledge culturally situated practices, and interventions anchored in local frameworks was emphasised [22,24,31,32,33,38,41,43,44,45,46,51]. These practices ranged from the spiritual [27] to narrative-based interventions [24,53].

#### 3.2.2. Community-Based Creative Practices

Community-based, creative therapy practices were used as both a curative and a resilience builder [29,50,54,55]. These interventions enabled affected communities to rebuild relationships, share experiences, and articulate, through creative mediums, their struggles and triumphs [28,31,35,40].

Community identity, often significantly impacted in post-disaster contexts, was also discussed as being critical in rebuilding communities [30,53], as was the re-establishment of trust [48] and embedding local knowledge in decision-making, and, hence, enhancing community viability [39,49].

#### 3.2.3. Community-Led Practices

Seven studies [21,26,28,38,39,40,47] discussed how improvement in community recovery was greater when the recovery arrangements were handled by the community in which the initial disaster occurred. Community-led practices and interventions require a different approach from those generally enacted by stakeholders in the institutional and political context such as emergency services and the government.

### 3.3. Leading and Leadership

Interlinking practices and interventions that are community-led is the theme of leading and leadership. Twenty of the included studies discuss the role and influence of leadership and leading in both facilitating and restraining recovery and healing for communities post-disaster. These aspects are discussed in two sub-themes: the criticality of trust in leadership and how a lack of effective community leading is a barrier to recovery and healing.

#### 3.3.1. Trust in Leadership

Trust in leadership during recovery is acknowledged as a crucial component of complex decision-making [56]. It is unsurprising then that a pre-existing connection between leadership and community is reported to facilitate rebuilding. In particular, processes and structures already familiar to people were argued to reduce anxiety [47,48,51]. In part, this is through facilitating access to previously safe spaces, which enables collective memory making [51]. While formal supports are imperative, informal supports via community leadership are equally so. Leadership embedded within the community has the capacity to provide culturally and geographically appropriate responses to the mental health and spiritual needs of local people, with community values rooted in storytelling, social bridging, building, and bonding [21,28,30,41,43,49].

#### 3.3.2. Barriers to Recovery

Conversely, in instances when the community did not lead recovery, marginalised groups were excluded from crafting and implementing responses [32,37]. Community concerns were not considered, for example, in the rebuilding process [32], and bureaucratic barriers frustrated recovery [47]. These barriers were illustrated in eight studies [29,32,33,35,39,40,43,55]. Equally, when impacted communities led their own recovery, the collective was empowered to create new assets, determine their own directions, and set their own priorities [24,26,41].

In summary, leaders having a good working knowledge of both the local conditions and the intricacies and complexities of the broader context in which both disaster and recovery processes are involved is more than just beneficial, it is crucial for improved outcomes [23].

### 3.4. Restraints to Healing and Recovery

Twenty studies identified restraints to healing and recovery [21,22,23,26,28,29,30,31,32,37,38,40,41,43,44,47,48,49,51,55]. Restraints broadly fall into three types, those associated with a sense of loss, feelings of alienation, and, finally, severed connection. These negative practices work together to create disenfranchisement and a sense of hopelessness.

#### 3.4.1. Loss and Disempowerment

Loss is discussed in relation to an eroded sense of control, decision-making, personal agency, and access to previously familiar and valued spaces [28,30,33,51]. Grief [23] and hurt [22] are evidenced in Clissord and colleagues’ paper [51] when the destruction of natural and built resources meant people questioned their ability to survive. Loss and disempowerment were also felt when cultural practices, such as collective crafting [40], or minority groups were ignored or dismissed in top-down government and social service agency management practices [22,32,40]. Institutional racism was found to prevent people from seeking a role in their own destiny and healing [38]. These practices that amplify loss and disempowerment demonstrate the criticality of context—how responses to and recovery from disasters are always situated and emerge in specific social, political, economic, environmental, and historical contexts.

#### 3.4.2. Sense of Alienation

Leadbeater and colleagues [26] discussed this sense of alienation in relation to governmental versus community measures of recovery, noting that, in spite of a commitment to a holistic view of recovery (Victoria Bushfire Reconstruction and Recovery Authority [57], matters pertaining to rebuilding infrastructure were most commonly used as key indicators of success, not all-inclusive recovery [48,49].

Risks, such as revictimisation [47] and detrimental mental health impacts from procedural injustices [21], or forced migration away from family and ‘place’ (home) [51], resulted in disenfranchisement. Such experiences commonly led to calls for a shift in approach to co-shared or community-led [37,43].

#### 3.4.3. Connectivity to Place

Connectivity to place, the strength that forms with collective identity, is apparent. Silver and colleagues [28] reinforce a sense of place and its fundamental importance in disaster recovery. Place is a strong theme within the Western discourse of geography, such that Vallance [41] asserts place is an obvious starting point for community formation post-disaster, demonstrating a clear healing role for the natural environment. Linton [55] and Block and colleagues [23] argue that facilitating a connection to the natural environment, through community activity, is a critical factor influencing disaster recovery, reviving a sense of home, a safe place, and sanctuary, which can engender a sense of hope and create the conditions of possibility for restitution and healing [55].

Notwithstanding the dominant view that disenfranchisement of people post-disaster is a barrier to recovery and that place and connectivity are crucial, the concept of Country and its role in healing is not recognised. Linton [55] and Block and colleagues [23] do discuss the role of the natural environment; however, no paper extends this approach to the holistic concept of Country and its role in mitigating limited Anthropocentric practices.

### 3.5. Strength-Based Approaches

Strengths-based approaches were a prominent theme evident in fourteen papers [22,26,33,38,39,40,41,43,44,47,48,49,50,51], which identified the importance of leveraging existing assets, not taking a deficit focus, but rather focusing on post-traumatic growth and honouring and holding trauma [33,40,49,51]. This theme is discussed in three sub-themes: leveraging community resources; the impacts of disaster on post-traumatic stress; and the role of community organisations.

#### 3.5.1. Leveraging Community Resources

Strengths-based approaches to recovery and healing focus on leveraging existing community resources and capabilities to reclaim power and enhance resilience against future disasters [22,33,49]. Strengths-based approaches engage and collaborate with the community, recognising the importance of time to facilitate answers to problems that address the nuanced needs of each community by building on existing skills and tools [26,38]. Taking a deficit approach restrains both communities’ ability to utilise their strengths and trust, which is built through authentic participation and engagement [39,40]. Moreover, when Indigenous knowledges and practices were prioritised throughout the process, communities were enabled to maintain a focus on their strengths [22,33]. Tamasese and colleagues [43] show that post-disaster responses need to be embedded in the values of the impacted communities, such as strengths-based, fun, and interactive, as well as using appropriate metaphors as a way of engaging participants in healing processes.

#### 3.5.2. Impacts of Disaster on Post-Traumatic Stress

Effectively building a community post-disaster requires an understanding of the impact of the disaster on post-traumatic stress for the individuals and community [33,49]. In the period of rebuilding, there is a need to pay attention to the person and their individual and collective stories, particularly for vulnerable groups, as this offers the possibility for positive change [38,40,41,51]. One modality offered to address post-traumatic stress was yoga. For instance, Heinz and colleagues [44] found it was an effective trauma-informed healing strategy.

The generosity of the community and the capacity of individuals to volunteer their time and resources are significant in the rebuilding phase [47,50]. The knowledge and wisdom, for example, within the community underpins the functioning of daily living [33]. Such self-efficacy was evident in the literature where the community utilised the diverse skills and pre-existing links that were present in their community, enabling problem-solving and sustainability to be recognised [41,48,49].

#### 3.5.3. Role of Community Organisations

Finally, the central role of community organisations and First Nations community-controlled organisations in enabling the implementation of strengths-based approaches to healing and recovery was evident in the literature [22,38,43,49]. For example, Storr and colleagues [49] demonstrate how the Broadmoor Improvement Association (BIA) drove community healing and recovery following Hurricane Katrina by adopting a strengths-based approach that valued local knowledge and expertise and successfully lobbied to access external funding. By ‘rebranding’ the community as one of strength, activity, and revitalisation, Broadmoor was not only rebuilt but able to attain BIA’s mottos of “Broadmoor Lives-in the Heart of New Orleans” and “better than before’ to emerge as a more robust, active, and unified community. 

### 3.6. Holistic Conception of Well-Being

Twelve papers included a conception of well-being that went beyond a single, psychological dimension. Two sub-themes evident in the papers that play important roles in enhancing social and emotional well-being are embodied practices and religion and spirituality.

#### 3.6.1. Embodied Practices

Some papers demonstrated that embodied practices such as yoga [44] or arts practice and associated gatherings to share experiences of disaster [32,40] were helpful for social and emotional well-being and healing. Traditional community conceptions of holistic well-being from around the world that encompassed physical, spiritual, emotional, social, and aesthetic dimensions [38] were evident in New Zealand Māori views [43]; in Samoan views [51]; in perspectives from Vanuatu [27]; from Alberta, Canada; and in Yolŋu views from Galiwin’ku, Australia [22].

Within this holistic view of well-being, there was recognition that connection to the environment influenced well-being, with several studies finding that attachment to the natural environment caused grief from seeing the loss of familiar landscapes and wildlife as a consequence of disaster [22,23,28,30]. Block and colleagues [23] also illustrated how this connection was also protective, with strong bonds to the natural environment correlated with less distress, depression, and PTSD, and greater levels of resilience and life satisfaction. 

#### 3.6.2. Religion and Spirituality

Religion and spirituality were found to be linked to well-being in seven studies [22,27,35,38,43,47,51], with benefits both from faith in a higher being contributing to resilience [22,47,51] and from community practices linked to faith enhancing healing and recovery [38,43]. Lalani and colleagues [27] found in the midst of physical losses people experienced metaphysical losses of identity, meaning, and purpose which affected well-being across physical, economic, spiritual, and psychosocial dimensions. In the face of these losses, spirituality was found to be a protective factor for both resilience and recovery, providing meaning, endurance, love, belonging, hope, peace, and gratitude. 

## 4. Discussion

The themes articulated in the findings section align with Hobfoll and colleagues’ [58] seminal work, which articulated the following critical elements of mass trauma intervention: (1) a sense of safety, (2) calming, (3) a sense of self-efficacy and community efficacy, (4) connectedness, and (5) hope. This review expands this work by focusing more attention on collective healing and recovery and adopting a Country-centred conception of community that is inclusive of humans, other than humans, and their entwined relationships.

**Figure 2:**
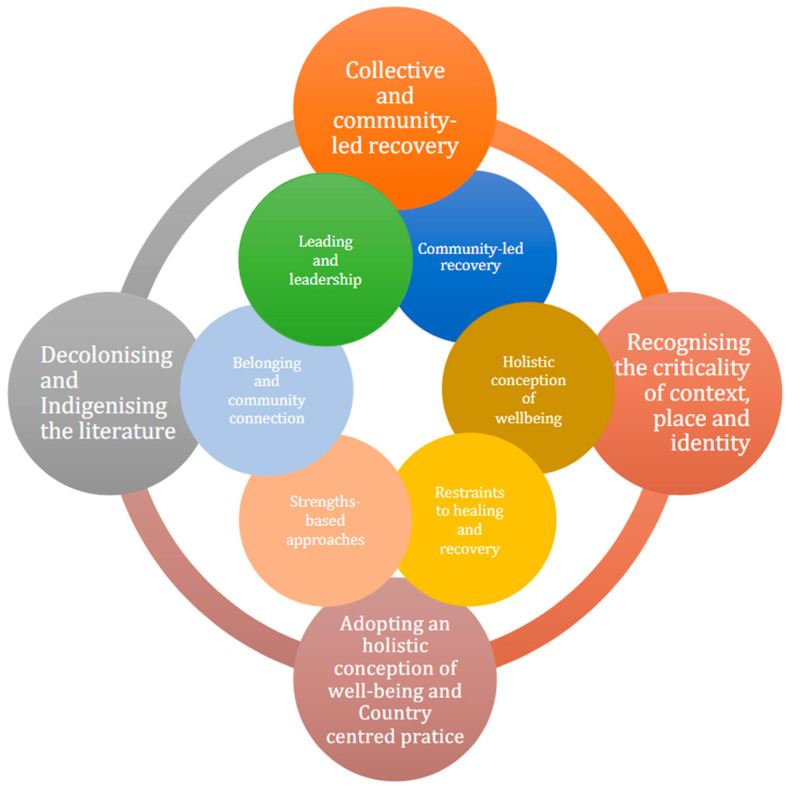
Key practices.

Four narratives were iteratively developed from the findings, foregrounding the key practices evident in the review: (1) collective and community-led recovery; (2) recognising the criticality of context, place, and identity; (3) adopting a holistic conception of well-being and Country-centred practices; and (4) decolonising and Indigenising the literature (Figure 2).

### 4.1. Collective and Community-Led Recovery

The disaster literature focused on recovery is dominated by interventions aimed at the individual and their mental health [59]. The entanglement between individual and community recovery and the practices that enhance and hinder collective recovery is much less evident. This is perhaps surprising, given the evidence that a strong sense of belonging, social connections, and participation in community groups are linked to better mental health outcomes [60,61]. Indeed, recovery from mental health issues post-disaster has been shown to require community-level interventions [62,63] rather than relying solely on individualistic approaches.

Collective recovery enables communities and individuals to manage their own recovery process, particularly when community development principles, whereby communities control their own pathway through identifying needs, strengths, and barriers, and designing strategy [64] underpin recovery practices and interventions. Working with these principles recognises that the community is best placed to develop strategies that suit them. Whilst pre-existing support networks were recognised in this review [21,22,29,43], a sense of belonging to a community and the comfort garnered from it [21] was less explored.

The papers reviewed provide evidence that community-led interventions are robust [29,36,46,51], with a focus on longer-term community-wide recovery [26,36,47]. Community-led interventions work with strengths and capabilities [65] as the preferred response for their ability to revive community abilities [29,32,33,35,39,40,43]. Evidence that outcomes are further enhanced when recovery is managed directly by those impacted was found in most studies [21,24,26,28,38,39,40,41,47].

Designing and delivering care that is contextually appropriate and responsive requires a deep knowledge of local relationships, resources, and events [66]. Within this framework, the contribution that trusted leadership lends to viable recovery [23,67] is vital. Leadership embedded in the community can build trust and empower people [56,68,69] through appropriate responses to the diverse needs of local communities. This is particularly the case when structures and processes familiar to people pre-disaster are entwined with the recovery process [47,48,51].

These practices of collective and community-led recovery work together to create a sense of belonging, a sense of place, a sense of safety, and a sense of hope for the future. Accordingly, these are key indicators of well-being and community connectedness [70] and are crucial to strengthening healing and recovery post-disasters. Overall, the key narrative to emerge here is the strength of the collective recovery and that community-led recovery must be prioritised and enabled to ensure more sustainable outcomes. 

### 4.2. Recognising the Criticality of Context, Place, and Identity

Responses are inevitably situated in relational, interdependent, and interconnected contexts that frame how recovery will progress. This review finds that when local culture and context are excluded, it is easy for local, particularly marginalised, voices to be unheard [32,37]. Communities facing disadvantage are disproportionately impacted, as losses do not mean the same thing to people living with security as they do to people living with precarity. These inequities and inequalities in recovery for, or exclusion of, marginalised groups, were evidenced in [32,37,38,47]. A top-down response, where outside agencies tend to implement ‘one size fits all’ disaster relief strategies, can create an unproductive, potentially hostile, atmosphere of mistrust, alienating locals and their situational knowledge [65,71]. The evidence from this review suggests the capacity of the community to drive recovery must be harnessed. Accordingly, outside recovery agencies need to be guided by the local knowledge and capabilities of the community [72].

This review also finds the importance of practices that are situated in place and identity. Restraints to healing are often couched in the language of loss of the familiar and previously valued, such that a sense of agency over individual and community identity is significantly eroded [28,30,33,51]. There is a critical link between the recovery of a sense of community and strengths-based community rebuilding [30,53]. Evidence also suggests that integrated communities are better situated to recover due to their social capital [42,48,49]. In sum, recovery practices that work with and build on existing social capital, by nurturing connectedness rooted in local practice and values, have a significant role in disaster response programs [33,36,45].

### 4.3. Adopting a Holistic Conception of Well-Being and Country-Centred Practice

Conceptions of well-being exceeding one psychological dimension were evidenced in a number of studies reviewed [32,40,44] and featured in those studies that focused on traditional, holistic notions of well-being [22,27,38,43,51].

Spirituality and religion were positively linked to well-being [22,27,35,38,43,47,51]. Faith is shown to enhance resilience [22,47,51] and, in particular, faith-based community practices contribute to healing and recovery [38,43].

There was some evidence of how connection to the physical environment contributes to enhancing well-being [22,23,28,30,41]. This improvement in well-being was particularly the case where community interventions were specifically aimed at nurturing a connection to natural places [23,55]. Notwithstanding, none of the studies extended the recognition of nature and human interaction to the holistic concept of Country, where Country is known as ‘a living entity…with a consciousness and will toward life’ [9]. 

A holistic approach to well-being is argued to be one of six globally recognised Indigenous healing elements [73,74], where the connectedness of the physical, mental, emotional, spiritual, and social aspects of life is underpinned by relationship, place and Country. In the Western worldview, humans have primacy over all non-human entities. On the contrary, a First Nations approach is founded on human and non-human relationships and connection to Country, which embodies human and non-human reciprocity, such that well-being underpins all aspects of life [75]. A Country-centred view places humans as part of the broader ecosystem within relational and dynamic networks. Country is seen “in oneness and living”, where oneness changes our viewing lens so that we no longer think of ‘environment’ as separate from ourselves, but instead recognise that we are part of the natural world; “Country is self, self is Country” ([76] (p. 19).

Our continued reliance on a system that mandates humans as separate, as the “privileged overseer” ([76] (p. 3) of nature, fails to leverage advantage from alternate Country-centred methodologies, those that encompass non-human and human entities and the connectivity amongst them. The key message here, as highlighted by Quinn and colleagues [73], is that holistic healing, which is embedded in Indigenous practices, offers insights into increasing disaster challenges and thus has a contribution to make in future recovery efforts.

### 4.4. Decolonising and Indigenising the Literature

In the context of permacrisis, there is an urgent need for the incorporation of First Nations’ knowledges and practices in this space so that alternate ontologies, epistemologies, and methodologies are recognised. Decolonisation must go beyond recognition to ensure the participatory formation of research objectives and methodologies that consider those impacted and are respectful of the most vulnerable sectors of society [77]. First Nations’ scholarship was represented in the literature studied [22,38,43,51], adding valuable and timely influence to the discussion. Nevertheless, it is noted that these papers were scarce and framed by Western knowledge systems.

A continued focus on Western hegemonic views of ‘nature’ as subordinate and separate, which prioritises human life and the associated formed environment over a Country-centred approach, must shift. In the context of the Anthropocene and permacrisis, this shift is a time-critical issue. Accordingly, First Nations’ knowledges and healing practices need to be foregrounded in disaster recovery.

## 5. Strengths and Limitations

A strength of this systematic review is that the authors are an interdisciplinary team of Aboriginal and non-Aboriginal researchers, most of whom live in regional and rural communities across the Southeast Coast of Australia that were impacted by and are recovering from the devastation of 2019–2020 Black Summer Bushfires. Accordingly, they have lived experience of the phenomena that are the focus of this review. Another strength is the range and number of countries (12) represented in the studies that include low-, middle- and high-income countries. A related limitation of this review is that it restricted papers to those published in English. Accordingly, relevant studies published in other languages were not included. Another limitation of this review is that it focused on the recovery stage of experiencing disaster, and thereby did not specifically include search terms such as resilience. As a result, this review may have excluded some relevant studies. Crafting the review question and selecting the keywords in a different way may result in different search outcomes. Additionally, the variability in study quality, measurement tools, and study designs across the included articles restrains the generalisability of findings. 

## 6. Conclusions

In summary, this systematic review adopted an Aboriginal-informed Country-centred notion of community underpinned by a social practice theoretical approach, thereby, contributing to an expanded conception of community and focusing more attention on collective healing and recovery. This review established a range of practices and interventions that support and/or hinder healing post-climate-related disasters for communities and the environments within which they live. Collective and community-led interventions were found to harness local knowledge, networks, and expertise, which improves the dissemination of resources and enables healing and recovery efforts to be tailored to the specific needs of communities. Such an approach supports a more inclusive and participatory approach to long-term recovery.

### 6.1. Future Research Recommendations

Given the identified gaps multiple recommendations can be offered for further research. First, further studies are required to articulate the specific practices and mechanisms that are effective in enhancing community social capital and resilience. Second, there is a need for further research into practices and interventions that address inequities and inequalities and prioritise the experience and voices of marginalised communities. Third, there is a need for research that investigates the links between individual healing and community recovery. Here, empirical examples of how First Nations’ knowledge and healing practices may be utilised to enhance community recovery would be helpful. Fourth, disaster studies should further question their grounding in Western-centric, humanist, and capitalist logics, which promote human exceptionalism and actively decolonise and Indigenise disaster research. Studies on responses to disasters that protect and nurture human and organic biodiversity are urgently required. Finally, given that we are living in a permacrisis with increasing exposure to climate-related disasters, further research is needed into enhancing recovery, healing, and resilience in the context of consecutive and cumulative disasters.

### 6.2. Recommendations for Practice

There are several recommendations for practice identified from this review. There is a scarcity of evidence on the practices and interventions that enhance collective healing and well-being for communities. There is a pressing need for Country-centred intervention programs and practices focused on the community level. Government and disaster recovery agency policies and practices that tend to centralise decision-making require a change to enable community-led recovery approaches to be implemented. Such approaches need to prioritise the active involvement of local communities and recognise that communities are the first responders and have valuable local knowledge, networks, and resources that are crucial for effective healing and recovery. Involving the community in response to disaster can be healing in itself, as such involvement brings community members together and can assist people to regain a sense of agency and control over their lives.

Disasters highlight, expose, and increase the social, cultural, economic, and health inequalities that are embedded in our societies. Accordingly, government and disaster recovery agencies need to develop more inclusive and participatory recovery approaches and practices that acknowledge the needs of diverse communities.

The deficit discourse that dominates much disaster recovery practice in relation to marginalised populations, including First Nations communities and communities struggling with poverty, needs to be replaced by a strengths-based discourse to enhance the possibility that culturally situated, place-based recovery solutions may flourish.

## Figures and Tables

**Table 1:** Example of search string.

**OVID Medline** 1: (practice* or Practice or intervention* or program or recover* or healing*).mp. [mp=title, abstract, original title, name of substance word, subject heading word, floating sub-heading word, keyword heading word, organism supplementary concept word, protocol supplementary concept word, rare disease supplementary concept word, unique identifier, synonyms] (3628973) 2: (natural disaster or Natural Disasters or (natural adj3 disaster) or wildfire or (wild adj3 fire) or (bush adj3 fire) or bushfire).mp. [mp=title, abstract, original title, name of substance word, subject heading word, floating sub-heading word, keyword heading word, organism supplementary concept word, protocol supplementary concept word, rare disease supplementary concept word, unique identifier, synonyms] (7278) 3: (‘Communities of Practice’ or community or Communities or (community adj3 environment)).mp. [mp=title, abstract, original title, name of substance word, subject heading word, floating sub-heading word, keyword heading word, organism supplementary concept word, protocol supplementary concept word, rare disease supplementary concept word, unique identifier, synonyms] (724960) 4: 1 and 2 and 3 (574) 5: practice.mp. or Practice Guideline/ (1135305) 6: (intervention* or program*).mp. [mp=title, abstract, original title, name of substance word, subject heading word, floating sub-heading word, keyword heading word, organism supplementary concept word, protocol supplementary concept word, rare disease supplementary concept word, unique identifier, synonyms] (2135354) 7: (recover* or healing*).mp. [mp=title, abstract, original title, name of substance word, subject heading word, floating sub-heading word, keyword heading word, organism supplementary concept word, protocol supplementary concept word, rare disease supplementary concept word, unique identifier, synonyms] (1005437) 8: 5 or 6 or 7 (3921243) 9: natural disaster.mp. or Natural Disasters/ (2322) 10: (bush fire* or bushfire*).mp. [mp=title, abstract, original title, name of substance word, subject heading word, floating sub-heading word, keyword heading word, organism supplementary concept word, protocol supplementary concept word, rare disease supplementary concept word, unique identifier, synonyms] (438) 11: (wildfire or wild fire*).mp. [mp=title, abstract, original title, name of substance word, subject heading word, floating sub-heading word, keyword heading word, organism supplementary concept word, protocol supplementary concept word, rare disease supplementary concept word, unique identifier, synonyms] (1953) 12: (((((natural adj3 disaster*) or wild) adj3 fire*) or bush) adj3 fire*).mp. [mp=title, abstract, original title, name of substance word, subject heading word, floating sub-heading word, keyword heading word, organism supplementary concept word, protocol supplementary concept word, rare disease supplementary concept word, unique identifier, synonyms] (171) 13: 9 or 10 or 11 or 12 (4663) 14: communit*.mp. or Residence Characteristics/ [mp=title, abstract, original title, name of substance word, subject heading word, floating sub-heading word, keyword heading word, organism supplementary concept word, protocol supplementary concept word, rare disease supplementary concept word, unique identifier, synonyms] (748309) 15: (community adj3 environment).mp. [mp=title, abstract, original title, name of substance word, subject heading word, floating sub-heading word, keyword heading word, organism supplementary concept word, protocol supplementary concept word, rare disease supplementary concept word, unique identifier, synonyms] (1645) 16: 14 or 15 (748309) 17: 8 and 13 and 16 (365)

**Table 2:** Inclusion/exclusion criteria.

	Relevant/Inclusion Criteria	Irrelevant/Exclusion Criteria	Exclusion Criteria for FULL TXT RX
Study Focus	Papers in English Papers referring to natural disasters or natural environmental events only Post-disaster practices or interventions (include if post-disaster is included in pre–post-disaster intervention) Focus on healing/recovery/ inclusive of environment (human-made and non-human as it relates to recovery) Focus on community recovery post-disaster	COVID-19 Global pandemics Pre-disaster Disaster preparedness/risk Disaster management Environmental pollution Terrorism/bioterrorism Technological disaster Conflict/war Humanitarian Crisis Climate change planning HIV/AIDS Wrong environmental impact (e.g., micro pods) Migration/immigration/refugee camp Nuclear disaster	Wrong study focus (e.g., not natural or environmental or post-disaster)
Data	Empirical studies Quantitative studies Qualitative studies	Prevalence studies Epidemiology/epidemiological studies	Wrong study data (e.g., prevalence study)
Participants	Humans and other than humans (e.g., animals, plants, reptiles, geographical places) Adults and children	Exclude animals if paper is focused only on a particular species and not inclusive of broader context	Wrong participants (e.g., humans or link to animals)
Interventions	Post-disaster interventions Post-disaster practices Healing practices Practices of recovery	Medical interventions (e.g., smoke inhalation, hypertension, symptom tracking, scale development, etc.) Mental health measures or tracking (e.g., depressive, PTSD symptom tracking without an intervention) Land or forest management focused on technical applications Fire management Pre-disaster interventions	Wrong interventions (e.g., no interventions or practices of recovery or healing)
Settings	Communities	Communities not impacted by disaster	Wrong setting (e.g., not community-based)
Publications	Research papers Research reports	Letters, editorials, commentaries, periodicals, conference abstracts, artworks, news updates, speeches Books reviews Books and book chapters Systematic reviews/other reviews Thesis dissertations Missing abstracts	Wrong publication (e.g., systematic review, book, or book chapter)
Language of Publication	English	Titles and abstracts in a language other than English	Not English manuscript
Publication Date	2002–2022	Pre-2002	Pre-2002

**Table 3:** Study characteristics.

Author/Year	Country	Aim (Summary)	Design	Target Population	Sample	Intervention Type	First Nation Authors
Akbar et al. 2018	Pakistan	Impact of social capital on recovery.	Quantitative	Individual	N = 450 residents	Not stated	N
Ali et al. 2021	Australia	Develop a holistic and all-hazards Indigenous community-based DRR theory.	Qualitative	Community	N = 20	Empowerment Strength-based	Y
Araki 2013	Japan	Capability building in the local community in actual disaster response and recovery.	Qualitative	Community	Not stated	Empowerment Strength-based	N
Banks et al. 2016	United States	Determine implications for community-focused interventions that may enhance recovery for vulnerable populations.	Mixed- Method	Community	N = 12	Not stated	N
Baumann et al. 2021	Nepal	Map community art in Kathmandu and expert perspectives about the relationship between community art and its impact on health, social cohesion, and community resilience.	Qualitative	Community	N = 19	Well-being	N
Becker 2009	India	Investigate the effectiveness of a community-based mental health initiative for survivors.	Quantitative	Community	N = 100	Resilience	N
Bender et al. 2015	Chile	Explore how everyday creative thinking, creative production, and intuitive experiences might have been experienced by survivors.	Qualitative	Individual	N = 97	Resilience	N
Block et al. 2019	Australia	Describe recovery trajectories in terms of mental health, well-being, and social connectedness.	Mixed-Method	Community	N = 25 (Qualitative) N = 597 (Quantitative)	Not stated	N
Chamlee-Wright et al. 2011	United States	Social capital aids in post-disaster community recovery and redevelopment.	Mixed-Method	Community	N = 301	Resilience Strength-based	N
Clissold et al. 2021	Vanuatu	Displacement more broadly illustrates the human impacts of these disasters.	Qualitative	Individual	N = 8	Empowerment Well-being	Y
Cox & Perry 2011	Canada	Discourse of disaster recovery and the social–psychological processes that were constituted by and constitutive of this discourse.	Qualitative	Community	N = 4	Other—discourse analysis	N
Dionisio & Pawson 2016	New Zealand and Japan	Overview of the two disasters, their contexts and key issues, and analyses of community-driven projects.	Qualitative	Community	N = 2	Resilience	N
Easthope & Mort 2014	United Kingdom	How people recover from disasters, so that the recovery itself becomes a form of social change.	Qualitative	Community	Not stated	Resilience	N
Harms et al. 2021	Australia	Focused on two key areas in medium- to high-affected communities, relating to community members’ perceptions of (1) what caused the biggest problems for them in their recovery and (2) what was the most useful support.	Qualitative	Community	N = 811	Well-being	N
Harvey et al. 2007	USA	Measure the mental health outcomes for participants in the Hurricane Choir.	Mixed-Method	Community	N = 127	Other—choir	N
Heinz et al. 2021	USA	Inform the science and practice of disaster mental health outreach for other communities.	Mixed-Method	Community	N = 160	Other—Mind Body Yoga and SPR	N
Johnston et al. 2012	New Zealand	Role of community participation in reducing anxiety and trauma in communities during two New Zealand earthquakes.	Mixed-Method	Not clear	N = 160	Resilience	N
Ku & Dominelli 2018	China	Marginalised groups be empowered and encouraged to participate in community design, planning, and building processes.	Qualitative	Community	Not stated	Empowerment	N
Ku & Ma 2015	China	Build individual and community capacity for post-disaster social reconstruction in the affected area through the action research process.	Qualitative	Community	Not stated	Empowerment	N
Lalani et al. 2021	Canada	Explored various meanings and concerns, along with tools and strategies that helped to nurture spiritual resilience and well-being among residents.	Qualitative	Community	N = 30	Other—spiritual	N
Leadbeater 2013	Australia	Importance of locally-endorsed community leaders in the complex, post-disaster environment.	Qualitative	Community	N = 30	Resilience	N
Linton 2017	Nepal	Implementation and use of art therapy in the context of emergency and immediate post-emergency phases after a natural disaster.	Qualitative	Community	Not stated	Other—art therapy	N
Mann et al. 2021	Japan	Explore the well-being of displaced residents in order to gain an understanding of the challenges they face, successes in overcoming these challenges, and potential future obstacles.	Qualitative	Community	N = 380	Resilience Well-being	N
Mohr 2014	Peru	Determine whether the reported positive effects of an art therapy intervention continued over long-term recovery.	Qualitative	Community	N = 11	Empowerment Other—art therapy	N
Onstad et al. 2013	USA	Community sustainability after natural disasters depends on adaptive capacities of individuals, families, and businesses.	Qualitative	Community	N = 44	Other—life story	N
Osofky et al. 2018	USA	Describe the development of the St. Bernard Parish YLP and evaluate if the program was associated with increasing self-efficacy and decreasing trauma symptoms.	Quantitative	Individual	N = 137	Empowerment Resilience Other—self-efficacy	N
Rawson 2016	New Zealand	Translational process from this research to the creation of Te Waioratanga, which loosely translates to mean the activation of wellbeing.	Qualitative	Individual	N = 32–48	Strength-based	Y
Rivera-Munoz et al. 2020	New Zealand	Development of a critical theoretical understanding of community resilience as an inherently political concept.	Qualitative	Community	N = 15	Resilience	N
Scott et al. 2017	Australia	Adaptation for Recovery project was evaluated to determine if the project contributed to community ideas of resilience.	Qualitative	Community	Not stated	Empowerment Resilience	N
Silver & Martin 2015	Canada	Examines the impacts of the F3 tornado that struck the commercial and civic heart of the community.	Mixed-Method	Individual	N = 35 N = 238	Not stated	N
Stofferahn, C.	Australia	Community characteristics allowed Northwood to recover so quickly from a natural disaster.	Qualitative	Community	N = 22	Resilience	N
Storr & Haeffele-Balch 2012	USA	Focus on the recovery efforts of Broadmoor after Hurricane Katrina.	Mixed-Method	Community	N = 300 N = 103	Resilience Social Capital	N
Tamasese et al. 2020	Samoa	Community-based single-session group intervention designed to address psychosocial needs of Samoan young people following a tsunami.	Qualitative	Community	N = 1295	Resilience Strength-based Well-being	Y
Tudor et al. 2015	New Zealand	The emergence of the crafting movement in Christchurch post-earthquake.	Qualitative	Community	N = 9	Other—crafting	N
Vallance 2011	New Zealand	Strategies three community groups in Christchurch used in their collective response to the first of the major earthquakes.	Qualitative	Community	N = 37	Not stated	N
van Kessel et al. 2015	Australia	Explore lay perspective, the nature of interventions that aid resilience in people who have experienced a disaster in the Australian context.	Qualitative	Individual	N = 19	Not stated	N

**Table 4:** MMAT.

		Screening Questions		Qualitative Studies					Quantitative Studies					Mixed-Method Studies				
First Author/Year	Study Type	S1	S2	1.1	1.2	1.3	1.4	1.5	4.1	4.2	4.3	4.4	4.5	5.1	5.2	5.3	5.4	5.5
		Are there clear research questions?	Do the collected data allow us to address the research questions?	Is the qualitative approach appropriate to answer the research question?	Are the qualitative data collection methods adequate to address the research question?	Are the findings adequately derived from the data?	Is the interpretation of results sufficiently substantiated by data?	Is there coherence between qualitative data sources, collection, analysis, and interpretation?	Is the sampling strategy relevant to address the research question?	Is the sample representative of the target population?	Are the measurements appropriate?	Is the risk of nonresponse bias low?	Is the statistical analysis appropriate to answer the research question?	Is there an adequate rationale for using a mixed-methods design to address the research question?	Are the different components of the study effectively integrated to answer the research question?	Are the outputs of the integration of qualitative and quantitative components adequately interpreted?	Are divergences and inconsistencies between quantitative and qualitative results adequately addressed?	Do the different components of the study adhere to the quality criteria of each tradition of the methods involved?
Akbar et al. 2018	Quantitative Descriptive study	Yes	Yes						Yes	Yes	Yes	No	Yes					
Ali et al. 2021	Qualitative study	Yes	Yes	Yes	Yes	Yes	Yes	Yes										
Araki 2013	Qualitative study	Cannot tell	Cannot tell	Cannot tell	Yes	Yes	Yes	Cannot tell										
Banks et al. 2016	Mixed-Method study	Yes	Yes											Yes	Yes	Yes	Cannot tell	Cannot tell
Baumann et al. 2021	Qualitative study	Yes	Yes	Yes	Yes	Yes	Yes	Yes										
Becker 2009	Quantitative study	Yes	Yes						Yes	Yes	Yes	Yes	Yes					
Bender et al. 2015	Qualitative study	Yes	Yes	Yes	Yes	Yes	Yes	Yes										
Block et al. 2019	Qualitative study	Yes	Yes											Yes	Yes	Yes	Yes	Yes
Chamlee-Wright et al. 2011	Mixed-Method study	Yes	Yes	Yes	Yes	Yes	Yes	Yes										
Clissold et al. 201	Qualitative study	No	Cannot tell	Yes	Yes	Yes	Yes	Yes										
Cox & Perry 2011	Qualitative study	No	Cannot tell	Yes	Yes	Yes	Yes	Yes										
Dionisio & Pawson 2016	Qualitative study	No	Cannot tell	Yes	Yes	Yes	Yes	Yes										
Easthope & Mort 2014	Qualitative study	No	No	Yes	Yes	Yes	Yes	Yes										
Harms et al. 2021	Qualitative study	Yes	Yes	Yes	Yes	Yes	Yes	Yes										
Harvey et al. 2007	Mixed-Method study	Yes	Yes											Yes	Yes	Yes	Yes	Yes
Heinz et al. 2021	Mixed-Method study	Yes	Yes											Yes	Yes	Yes	Yes	Yes
Johnston et al. 2012	Qualitative study	Yes	Yes											Yes	Yes	Yes	Yes	Yes
Ku & Dominelli 2018	Qualitative study	Yes	Yes	Yes	Yes	Yes	Yes	Yes										
Ku & Ma 2015	Qualitative study	Yes	Yes	Yes	Yes	Yes	Yes	Yes										
Lalani et al. 2021	Qualitative study	Yes	Yes	Yes	Yes	Yes	Yes	Yes										
Leadbeater 2013	Qualitative study	Yes	Yes	Yes	Yes	Yes	Yes	Yes										
Linton 2017	Qualitative study	Yes	Yes	Yes	Yes	Yes	Yes	Yes										
Mann et al. 2018	Qualitative study	No	Cannot tell	Yes	Yes	Yes	Yes	Yes										
Mohr 2014	Qualitative study	Yes	Yes	Yes	Yes	Yes	Yes	Yes										
Onstad et al. 2013	Qualitative study	Yes	Yes	Yes	Yes	Yes	Yes	Yes										
Osofky et al. 2018	Quantitative study	Yes	Yes						Yes	Yes	Yes	Cannot tell	Cannot tell					
Rawson 2016	Qualitative study	Yes	Yes	Yes	Yes	Yes	Yes	Yes										
Rivera-Munoz et al. 2020	Qualitative study	Cannot tell	Cannot tell	Yes	Yes	Yes	Yes	Yes										
Scott et al. 2017	Qualitative study	Cannot tell	Cannot tell	Yes	Yes	Yes	Yes	Yes										
Siver & Martin 2015	Mixed-Method study	Yes	Yes											Yes	Yes	Yes	Yes	Yes
Stofferahn, C.	Qualitative study	Yes	Yes	Yes	Yes	Yes	Yes	Yes										
Storr & Haeffele-Balch 2012	Qualitative study													Yes	Yes	Yes	Yes	Yes
Tamasese et al. 2020	Qualitative study	Cannot tell	Cannot tell	Yes	Yes	Yes	Yes	Yes										
Tudor et al. 2015	Qualitative study	Cannot tell	Cannot tell	Yes	Yes	Yes	Yes	Yes										
Vallance 2011	Qualitative study	Cannot tell	Cannot tell	Yes	Yes	Yes	Yes	Yes										
van Kessel et al. 2015	Qualitative study	Yes	Yes	Yes	Yes	Yes	Yes	Yes										
